# Contrasting responses of *Thermocyclops crassus* and *T. oithonoides* (Crustacea, Copepoda) to thermal stress

**DOI:** 10.1038/s41598-024-58230-4

**Published:** 2024-04-01

**Authors:** Kacper Nowakowski, Łukasz Sługocki

**Affiliations:** 1 Department of Hydrobiology, Institute of Biology, University of Szczecin, Wąska 13, 71-715 Szczecin, Poland; 2 Center of Molecular Biology and Biotechnology, University of Szczecin, Wąska 13, 71-715 Szczecin, Poland

**Keywords:** Freshwater ecology, Zoology, Ecology, Environmental sciences

## Abstract

Thermal tolerance is a critical factor influencing the survival of living organisms. This study focuses on the thermal resistance of copepod species, *Thermocyclops crassus* (Fischer, 1853) and *T. oithonoides* (Sars G.O., 1863), with overlapping distribution ranges in Europe. Short-term heat shock experiments were conducted to assess the thermal resistance of these copepods, considering various temperature increments and exposure durations. Additionally, the study explored the influence of heat shock on egg sac shedding, a vital indicator of population dynamics. Results indicate that widely distributed *T. crassus* exhibits higher thermal tolerance compared to narrowly distributed *T. oithonoides*, with survival rates varying under different heat shock conditions. Furthermore, *T. crassus* demonstrated a quicker response in dropping egg sacs in response to thermal stress, suggesting a potential adaptive mechanism for the survival of adults. However, rapid egg sac droppings pose high risks for eggs facing unfavorable conditions. *T. crassus*, inhabiting environments with greater temperature fluctuations such as the littoral and pelagial zones, exhibited better survival mechanisms compared to *T. oithonoides*, which predominantly resides in the pelagic zone. The findings have implications for understanding copepod responses to global warming and thermal pollution. This research contributes insights into the adaptive strategies of thermophilic copepod species and their ecological consequences.

## Introduction

Thermal tolerance is crucial for survival of any living organism^1^. Species exhibit varying degrees of tolerance to environmental factors, with death occurring if the tolerance limit of a particular species is exceeded^2,3^. The ability of a species to resist the new thermal conditions may determine how vulnerable it is to temperature increases brought on by climate change^4–7^. Along with long-term increases in temperature, other warming events could affect aquatic animals. The thermoelectric power sector is one of the principal contributors of freshwater thermal pollution^8^. The ability to withstand heat stress may help animals expand their geographic ranges or adjust to changing climatic circumstances.

**Figure 1:**
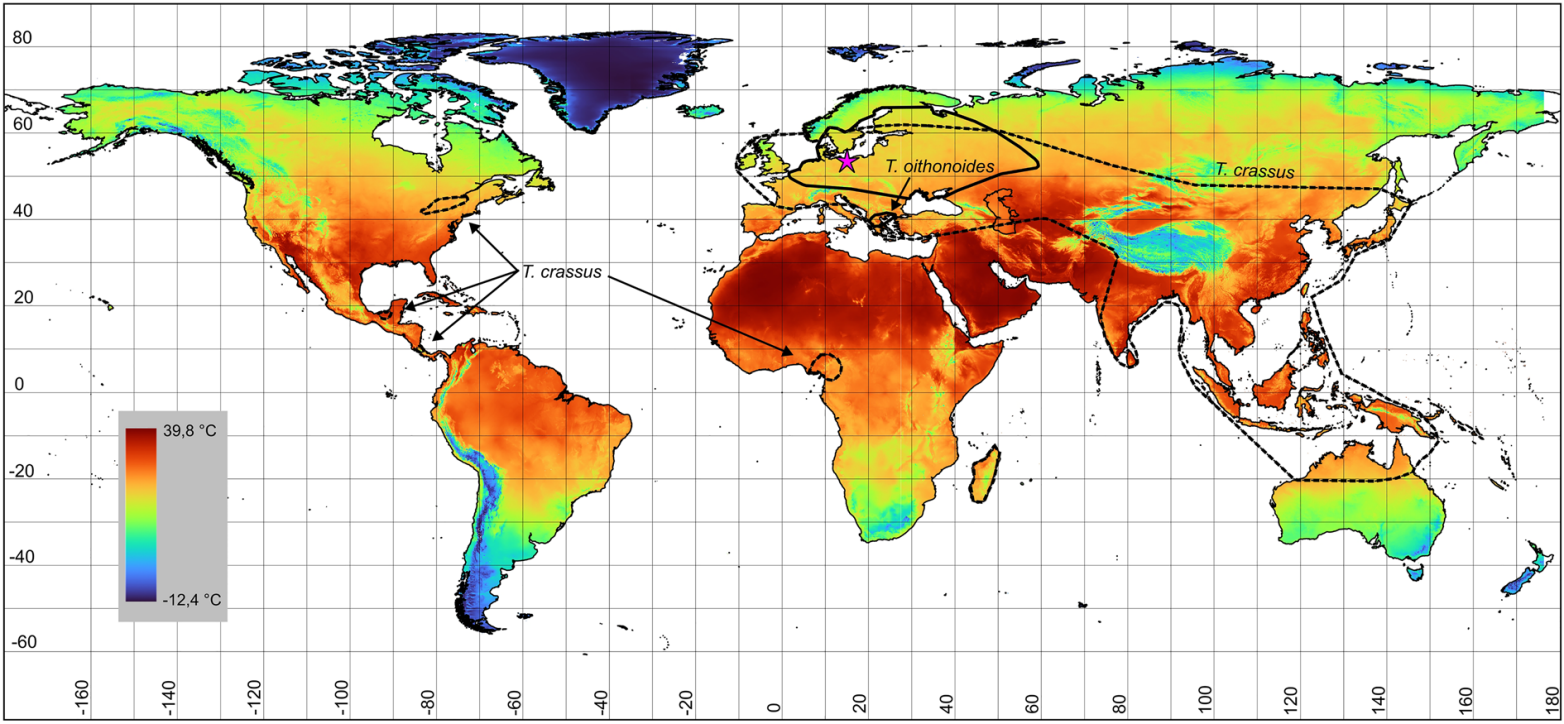
The map of the distribution ranges of *T. crassus* (dotted line) and *T. oithonoides* (solid line). The map for temperature refers to maximum temperature (°C) for globally the warmest month. The pink star indicates the location of copepod population sampling.

Copepod heat tolerance increases as annual temperature of their occurrence increases^9^. In order to better understand the thermal resistance of aquatic organisms, short-term shock may be used as a predictor of resistance to thermal stresses^10,11^. Previous work on thermal performance and thermal resistance mainly focused on marine and intertidal copepods, mostly related to Calanoida and Harpacticoida^9^. Studies on freshwater Cyclopoida in the context of thermal performance are rare. *Thermocyclops* Kiefer, 1927 (Crustacea, Copepoda, Cyclopoida) is a genus with a large variety of species that inhabit mainly tropics but occur also in temperate regions^12,13^. This genus is important component of zooplankton communities in many tropical and temperate water bodies^14–16^. *Thermocyclops crassus* (Fischer, 1853) is considered a cosmopolitan species because its range is dispersed across the globe while the range of *T. oithonoides* (Sars G.O., 1863) is restricted to Europe. The distribution ranges of *T. crassus* and *T. oithonoides* overlap across Europe (Fig. 1). *Thermocyclops* species found in tropical areas reproduce year-round, while species found in temperate zones reproduce in the warm season only^16^, therefore all known species in this genus are consider as thermophiles. *T. crassus* inhabits lakes, rivers, reservoirs, ponds, streams, marshes, typically related to pelagial and litoral zone^17^. *T. oithonoides* inhabits mainly pelagial zone of lakes, however is sometimes noted in large rivers and reservoirs^17,18^.

The aim of this study is to determine whether the current geographical distribution of *Thermocyclops crassus* and *T. oithonoides* reflects those species’ tolerance to temperature stress. To test the thermal resistance of copepods, experiments with various heat shock conditions and exposure duration were carried out. The impact of heat shock on egg sac shedding was also studied which is an additional indicator of population characteristics.

## Results

### Survival analysis

**Figure 2:**
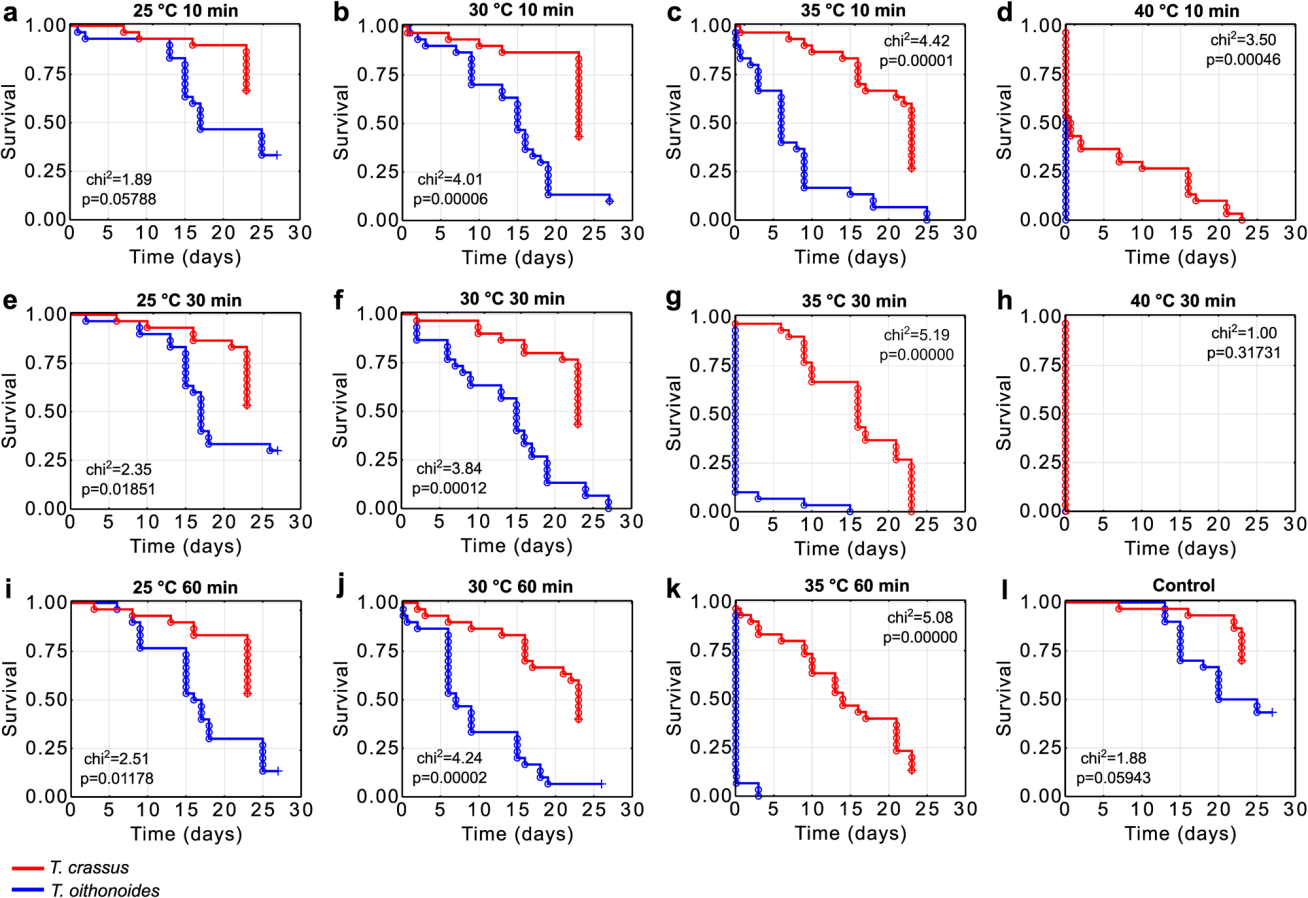
Kaplan–Meier survival curves for different exposure durations (**a**-**d** – 10 min; **e**–**h** – 30 min; **i**-**k** – 60 min; **l** – control) and thermal shock scenarios (**a**, **e**, **i** – 25 °C; **b**, **f**, **j** – 30 °C; **c**, **g**, **k** – 35 °C; **d**, **h** – 40 °C). The results of the log-rank test indicate statistical significance at a level of *p* < 0.05. Circles indicate deceased copepods, and crosses represent censored data.

In laboratory conditions, *T. oithonoides* proved to be a more sensitive to temperature stress than *T. crassus* (Fig. 2). Despite noticeable differences in survival under control conditions, these distinctions did not reach statistical significance (*p* > 0.05). Starting from the smallest temperature shock of 5 °C lasting for 30 min, a gradual divergence in the survival curves of the investigated copepods was observed. These differences escalated with the increase in thermal shock intensity and the duration of exposure to this stressor. At a 10 °C thermal shock lasting for 60 min, a substantial decrease in the survival of *T. oithonoides* was noted. Under these conditions, on the sixth day of the experiment, nearly half of the *T. oithonoides* population succumbed, whereas only 10% of *T. crassus* perished. With a 15 °C shock, these differences further amplified, and following a 30-min exposure, 90% of the *T. oithonoides* population was deceased, while over 90% of the *T. crassus* population remained viable. A brief 20 °C shock was survivable only by *T. crassus* individuals, though they did not endure prolonged exposure to this stressor.

### Egg sacs dropping

**Figure 3:**
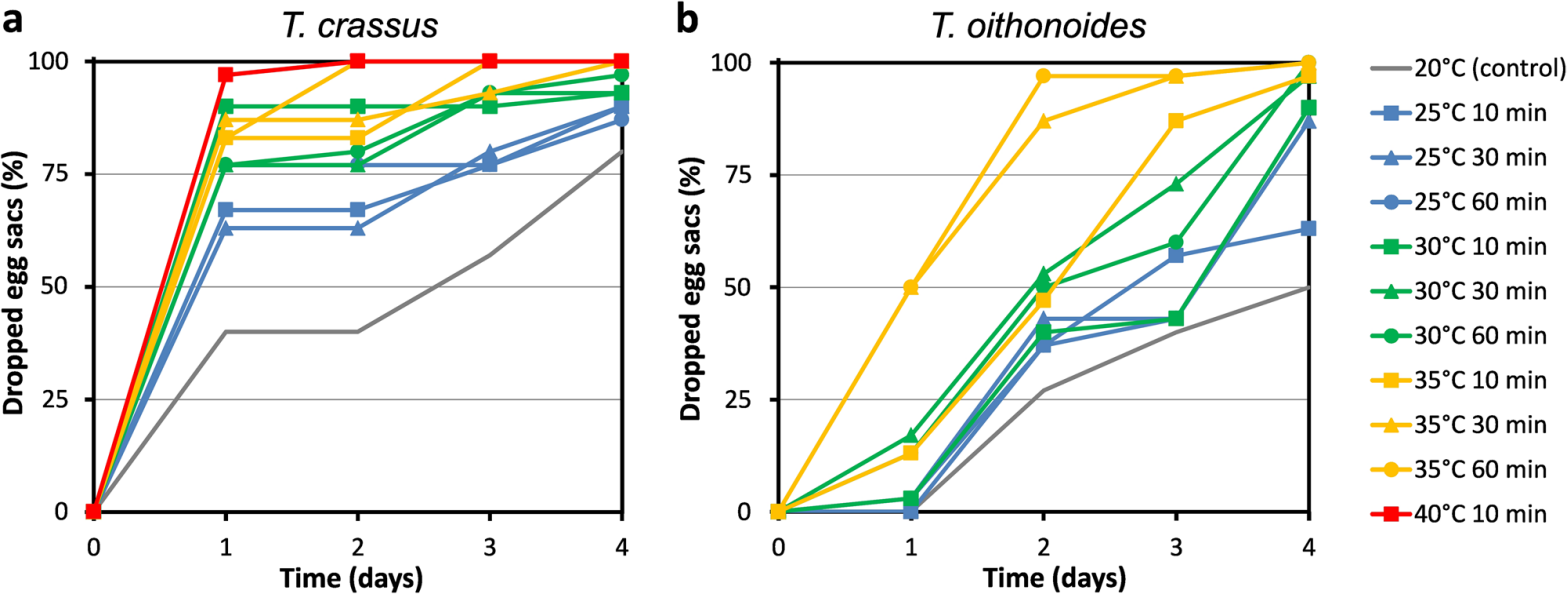
Dropped egg sacs by *Thermocyclops crassus* (**a**) and *T. oithonoides* (**b**) exposed to temperature shock for varying time periods. Only individuals that survived the initial 40 °C exposure on the first day of the experiment are included in the results.

In control conditions, *T. crassus* exhibited a faster egg sac dropping rate compared to *T. oithonoides* (Fig. 3). On the first day of the experiment under control conditions, none of the *T. oithonoides* individuals dropped egg sacs, while during the same period, 40% of the *T. crassus* population released egg sacs. Both investigated species demonstrated a response to thermal stress and its duration by releasing egg sacs. *T. crassus* exhibited a quicker response to thermal stress than *T. oithonoides*. On the first day of the experiment, at a + 5 °C shock, 63–67% of the *T. crassus* population dropped egg sacs, at + 10 °C, 77–90% dropped egg sacs, at + 15 °C, 83–87% dropped egg sacs, and at + 20 °C, 97% dropped egg sacs. Meanwhile, *T. oithonoides* at + 5 °C dropped egg sacs in 0–3% of the population, at + 10 °C, 3–17% dropped egg sacs, and at + 15 °C, 13–50% dropped egg sacs. Only on the second day of the experiment was a significant increase observed in the egg sac dropping by *T. oithonoides*. On the fourth day of the experiment, under control conditions, 80% of the *T. crassus* population dropped egg sacs, at + 5 °C, 87–90% dropped egg sacs, at + 10 °C, 93–97% dropped egg sacs, and at + 15 °C and + 20 °C, 100% dropped egg sacs. During the same period, in control conditions, 50% of the *T. oithonoides* population dropped egg sacs, at + 5 °C, 63–87% dropped egg sacs, at + 10 °C, 90–100% dropped egg sacs, and at + 15 °C, 97–100% dropped egg sacs. In cases where survival on the initial day of the experiment was 0, indicating that copepods did not survive beyond the first day, egg sacs were not dropped before their death.

The ANOVA results indicated that temperature was a decisive factor in relation to egg sacs dropping, while exposure time did not play a significant role in this case (Table 1). A stronger impact of temperature was observed for *T. crassus* (*p* = 0.0003) than for *T. oithonoides* (*p* = 0.0430).

**Table 1:** Results of two-way ANOVA using data for dropped egg sacs in *T. crassus* and *T. oithonoides* in relation to temperature shock values and exposure time.

	Df	Sum sq	F	*p*
*T. crassus*				
Temperature	2	0.1749	11.069	**0.0003**
Exposition	2	0.0085	0.5409	0.5844
Interaction	4	0.0096	0.3036	0.8691
Residual	27	0.2133		
Total	35	0.4064		
*T. oithonoides*				
Temperature	2	0.7523	3.5408	**0.0430**
Exposition	2	0.1572	0.7399	0.4866
Interaction	4	0.0546	0.1285	0.9707
Residual	27	2.8683		
Total	35	3.8325		

Df, degrees of freedom; Sum sq., sum of squares. Statistical significance is denoted in bold (*p* < 0.05).

## Discussion

Species may be able to withstand temperature extremes higher than those to which they are typically exposed^19^. Despite both *Thermocyclops* species being acclimated to the same thermal conditions and developing their populations in the same central European region, their responses to thermal stress differed. The limited studies on intraspecific variability in copepod temperature tolerance indicate that some copepods exhibit diverse responses to thermal stress, depending on the local conditions to which they have adapted^20–22^. Pereira et al*.*^21^, studying the mechanisms driving thermal adaptation in the intertidal copepod *Tigriopus californicus* (Baker, 1912), revealed significant variations in both genetic adaptation and developmental phenotypic plasticity among populations. Hence, the increased resilience observed in *T. crassus* may indicate genetic differences between the studied species, potentially affecting both acclimation capacity and/or developmental temperature. The results of our study suggest that the geographic occurrence range of *Thermocyclops* species may be impacted by their resistance to thermal stress; species like *T. crassus* that possess traits supporting its survival under conditions of thermal stress may be able to survive across a wide range of habitats.

In only a few taxonomic groups has the association between heat tolerance and latitudinal ranges of species been assessed^19^. In general, a species’ latitudinal range reflects its thermal tolerance, where heat tolerance corresponds to the peak summer temperature of its range^1^. However, temperature differences between regions at the same latitude are substantial. Latitude is a proxy indicator of temperature; hence, it does not always accurately reflect the thermal characterization of a region where a species occurs. The maximum temperature in the warmest months of the year served as our reference point. Maximum temperatures of locations where *T. crassus* has occur (according to species distribution presented in Fig. 1) are reaching 32 °C. Verbitsky et al*.*^23^, studying *T. crassus* populations in East Europe, concluded that the optimum thermal conditions for *T. crassus* are 25–30 °C, and a temperature of 34 °C is lethal for this species. We demonstrated that a high percentage of the population could survive temporary exposure to a temperature of 35 °C. Our results confirm the ability of *T. crassus* to withstand strong thermal stress, induced suddenly without acclimatization to conditions with elevated temperatures. This characteristic enables *T. crassus* expansion; therefore, this species is observed in many areas with a tropical climate. Despite *T. oithonoides* thermal preferences (this species occurs in Central Europe during the summer) suggesting a spread towards the equator, its range is limited to a maximum air temperature of 24.8 °C in Central Europe and 27 °C in South Europe. The limited ability of this species to survive thermal stress, on the one hand, and its narrow, restricted range associated with temperature, on the other hand, suggests that it is a thermophilic but also stenothermic species.

To better comprehend the enhanced thermotolerance of *T. crassus*, consideration should be given not only to the current distribution of species but also to migrations over the past few thousand years. It is speculated that during the last glaciation, *T. oithonoides* was present on the southeastern edge of the retreating ice sheet. After the glacier’s retreat, those niches were occupied by this species. In contrast, warmer refugia in Asia might have been inhabited by *T. crassus* (which suggests the current distribution of the species). Therefore, the colonization of Central European areas by *T. crassus* after the glacial retreat could have been carried out by populations of eastern origin that were previously adapted to higher temperatures.

*T. crassus* exhibits better-developed survival mechanisms under thermal stress. The genus *Thermocyclops*, most likely of tropical origin^24^, probably possesses mechanisms to mitigate excessive thermal stress. However, *T. oithonoides*, whose range is associated with temperate climates, has seemingly lost some of its heat stress mitigation potential. We hypothesize that this divergence is due to different habitat preferences. The littoral environment is more thermally variable due to the wind-driven water masses and the faster heating and cooling of shallower water layers^25^. *T. crassus*, which resides both pelagial and littoral habitats, is exposed to greater temperature amplitudes than *T. oithonoides*, which predominantly inhabits pelagial of lakes. The potential for *T. crassus* to competitively replace *T. oithonoides* is substantial, owing to the former’s broader range of thermotolerance. This observation aligns with research conducted on closely related marine cyclopoids, including the indigenous *Oithona nana* Giesbrecht, 1893 and the invasive *O. davisae* Ferrari F.D. & Orsi, 1984, as documented by Isinibilir et al*.*^26^.

Rahlff^27^, in a study of the molecular effects of heat shock on the species *Acartia tonsa* Dana, 1849 and *Eurytemora affinis* (Poppe, 1880), demonstrated that exposure to high temperature induces molecular changes, specifically an increase in the production of heat shock proteins, even in the absence of a visible reaction in copepods. The production of thermal proteins rises with increasing temperature, playing a role in maximizing the chances of surviving heat shock by repairing damage at the molecular level^28^. Low et al*.*^2^, investigating thermal stress in the widely distributed tropical copepod *Pseudodiaptomus annandalei* Sewell, 1919, revealed that maximal expressions of hsp70 and hsp90 protein genes occur at 32–33 °C. They found that maximum protein expression occurred 3 °C-3.5 °C above the mean water temperature (29.32 °C) of the copepod in the field. Individuals producing more thermal proteins can withstand greater thermal shock. Therefore, it can be inferred that in *T. crassus*, the production of these proteins may be higher than in *T. oithonoide*, resulting in greater thermal shock tolerance. Alternatively, *T. crassus* may have higher thermal limits and thus experience less stress than *T. oithonoides* at any given temperature, potentially resulting in lower expression of heat shock proteins. Further research on this variability at the molecular level is necessary to demonstrate whether *T. crassus* exhibits greater activity of proteins associated with the organism’s response to thermal stress compared to *T. oithonoides*.

The release of egg sacs in *T. crassus* was faster than in *T. oithonoides*, although both species were sensitive to thermal stress, leading to a more frequent release of egg sacs as the temperature increases. The ability for a quicker release of egg sacs may be an adaptation that enhances survival in unfavorable thermal conditions. Examples are known among other animals (eg. king penguin *Aptenodytes patagonicus* J. F. Miller, 1778) where females, subjected to strong stress, abandon eggs^29^. Moreover, in some crustaceans (crabs: *Lithodes santolla* (Molina, 1782) and *Paralomis granulosa* (Hombron & Jacquinot, 1846)) egg loss were observed in stress conditions^30^. There are examples in copepods where unattended eggs successfully hatched, especially in Calanoida^11,31^. Recent studies on copepod survival capabilities in challenging environmental conditions suggest that the release of eggs subjected to thermal shock could be a mechanism increasing not only the female’s chances of survival but also enabling the survival of the offspring. Bartholmeé et al*.*^32^, conducted an experiment to assess if the egg sacs of calanoids (*Eudiaptomus gracilis* (Sars G.O., 1863), *E. graciloides* (Lilljeborg, 1888)) and cyclopoids (*Cyclops abyssorum* Sars G.O., 1863, *Macrocyclops albidus* (Jurine, 1820)) passing through the digestive system of a fish can survive. They found that a total of 50–70% of the calanoid eggs and 11–29% of the cyclopoid eggs survived ingestion and gut passage. However, in our experiment, we did not monitor the hatching of young individuals from the released egg sacs. The shedding of egg sacs may serve as an adaptation for adult females to survive stressful conditions, as it enables quicker escape and reduces energy expenditure in case of increased effort^33^. Shedding egg sacs can present both opportunities and risks for the released eggs. It serves as an opportunity when the egg sacs fall onto favorable environmental conditions (e.g., well-oxygenated surfaces devoid of predators), while posing a risk if the eggs land in an unfavorable zone for their survival (e.g., oxygen-deprived zone or with limiting biological factors). However, this matter necessitates further research.

The obtained results also have implications for zooplankton assemblages affected by thermal pollution. Changes in abundance and zooplankton communities were investigated in cooling canals at the Konin and Pątnów power plants, Poland^34^. Tunowski^34^ demonstrated that a change as small as 7–8 °C leads to a decrease in the abundance of crustacean zooplankton ranging from 44 to 60%. In the case of the studied species, *T. oithonoides*, being a more sensitive species, has lower chances of surviving thermal shock compared to *T. crassus*. As each species has specific resilience to stressful conditions, communities under thermal pressure likely shift towards species characterized by greater resistance to thermal stress.

Richardson^35^ conducted research on the potential impact of global warming on zooplankton. The results obtained indicated that even a slight increase in temperature will causes a decrease in zooplankton abundance. Consequently, if climate change disrupts the zooplankton population, the induction of a short-term thermal shock associated with thermal pollution will lead to synergy of harmful conditions and reduction in copepod survival. As an organism capable of entering diapause, *Thermocyclops* possesses excellent abilities to survive adverse thermal conditions during the winter period (low temperature). In *T. oithonoides*, diapause occurs in copepodid stages IV–V during winter, either in sediments or in the pelagic zone^14^. However, in the case of climate changes leading to an increase in maximum temperatures, these thermal changes during the summer, may impact *Thermocyclops* to some extent. It is conceivable that an extension of the growing season due to global warming could lead to an elongation of the period where *Thermocyclops* reproduce. Nilssen and Wærvågen^14^ demonstrated, based on studies of *T. oithonoides* populations, that in the cold lakes in Norway, this species exhibited only one generation per year, while in warmer lakes, there could be two or three generations. Therefore it is likely that *Thermocyclops* will be a more constant component of Central European waters during the year. In addition to temperature, trophic status may also influence the rate of copepods development, accelerating it under adequate nutritional conditions. It seems that both thermal changes and increased eutrophication will favor an increase in the number of generations per year in *T. oithonoides*. With higher temperatures in the summer, both species may descend into deeper and cooler lake layers^14^. However, one of the threats to them could be local oxygen deficiencies preventing their migration^36^. Therefore, populations of *T. oithonoides* in shallow lakes appear to be the most vulnerable in the case of significant global thermal changes. Current changes seem to facilitate the further spread of *T. crassus* and other thermophilic copepod species. Our findings could be important in understanding the impact of global warming on thermophilic organisms.

## Methods

We selected two copepod species whose ranges overlap in some regions but have distinct climatic zone distributions^12,14^. This enables us to assess the thermal resistance of both species, assuming that both are adapted to the local thermal conditions. To assess the effect of short-term heat shock, we chose planktonic micro-crustaceans, *T. crassus* (Fischer, 1853), and *T. oithonoides* (G.O. Sars, 1863), which are closely related taxonomically and morphologically. The following four variants of thermal shock has been chosen: 25 °C (+ 5 °C), 30 °C (+ 10 °C), 35 °C (+ 15 °C) and 40 °C (+ 20 °C) in relation to the reference conditions (20 °C). Temperature change for copepods was instantaneous; individuals were not acclimated to the increased temperature. For all temperature variants, three exposure times were established: 10, 30, and 60 min. Individuals were subjected to heat shock only once at the beginning of the experiment. Both species were obtained from waters within the city of Szczecin, Poland. *T. crassus* was obtained (June 04, 2021) from the Oder river (53.427757N, 14.573041E) and *T. oithonoides* was obtained (June 04, 2021) from Głębokie lake (53.474399N, 14.478487E). The mentioned water bodies do not form thermal layers, and their temperatures are similar from the surface to the bottom, and both are eutrophic.

The water was heated in laboratory water bath (Microm, SB 80) with an accuracy of 0.1 °C. The vessels with water were placed in the bath, and after attaining a certain temperature, copepod individuals were introduced into the heated container for a defined period of time. Additional details about the experimental procedures are provided in the following paragraph. To monitor the accuracy of the temperature values, we used a multifunctional field laboratory device (Elmetron CX-401). The oxygen concentration in the heated containers was also controlled, but no significant decrease in oxygenation was observed. Thermal shock conducted on copepods has shown that the acclimation temperature has a large effect on the tolerance of individuals of a given species^10,27^. Therefore, in the experiment, individuals under similar thermal conditions were used by carrying out their acclimation. Twenty four hours before the experiment, all specimens were acclimated to the laboratory condition.

For each variant and exposure time, 30 individuals were gently selected and placed in three separate 120 ml containers (10 individuals in each container). For the experiment, we used only females: 360 specimens per taxon. We examined only adult females with egg sacs. This was because determining the sex of juvenile copepod (without stressing the animal) is difficult. After thermal shock was induced, crustaceans were placed under stable laboratory conditions at approximately 20 °C. To provide the crustaceans with optimal conditions during the experiment, all individuals were placed under the same lighting conditions (LED light, white color 6 W;16 h light/8 h dark). During the experiment *T. crassus* individuals were held in filtered water (mesh size: 100 µm) from the Oder river (at the time of copepod collection conductivity was 749 µS/cm, oxygen concentration was 6.9 mg/L, pH was 7.61, and the temperature was 19.5 °C). In *T. oithonoides* experiment, we used filtered water (mesh size: 100 µm) from Głębokie lake (at the time of copepod collection conductivity was 560 µS/cm, oxygen concentration was 7.43 mg/L, pH 7.31, and the temperature was 20.5 °C). Additionally, the individuals were fed regularly (1 µg of powder *Chlorella* sp. per container every alternate day). Before classifying the specimen as dead, its vital functions were checked twice under a microscope. Egg sac dropping was checked along with the assessment of adult survival. However, the morphology or development of the eggs in the egg sacs was not assessed. Adult *T. crassus* females had an average body size (mean and standard deviation) of 0.755 ± 30 mm and *T. oithonoides* of 0.708 ± 14 mm, calculated as the sum of the lengths of the cephalothorax, abdomen, and caudal furca.

The map of the distribution ranges was created based on the literature for *T. crassus*^13,15,37–48^ and *T. oithonoides*^14,49–54^. The map for surface temperature refers to maximum temperature (°C) data for 1970–2000 for globally the warmest month^55^(www.worldclim.org). Averaged spatial data for July were utilized to generate the map. A color gradient was applied to the resulting spatial data. The map was constructed using the World Geodetic System 1984 ensemble (EPSG:6326). The map illustrating the distribution of species in relation to thermal conditions across space was created using QGIS version 3.20.3. (https://www.qgis.org)^56^.

Survival analysis was conducted using the log-rank test, a non-parametric method for comparing survival distributions between groups^11,57^. Survival analysis was chosen for its ability to handle censored data and time-to-event outcomes. Chi-square and *p*-values (significance level set at α = 0.05) were computed to assess the significance of differences in survival curves. Additionally, we tested for differences in egg sacs dropped among species regarding the main factors in our experiment (temperature and exposure time) using a two-way analysis of variance (ANOVA). Before applying the statistical test, we checked the data distribution using descriptive statistics and histograms. We also assessed the homogeneity of variances with Levene’s test. All analyses were performed using Statistica 13.1 (StatSoft).

## Data Availability

The datasets generated during and/or analyzed during the current study are available from the corresponding author on reasonable request.
